# The Role of Intolerance of Uncertainty and Working Alliance in the Outcome of Cognitive Behavioral Therapy for Generalized Anxiety Disorder Delivered by Videoconference: Mediation Analysis

**DOI:** 10.2196/24541

**Published:** 2021-03-15

**Authors:** Gabrielle Marcotte-Beaumier, Stéphane Bouchard, Patrick Gosselin, Frédéric Langlois, Geneviève Belleville, André Marchand, Michel J Dugas

**Affiliations:** 1 Département de psychoéducation et de psychologie Université du Québec en Outaouais Gatineau, QC Canada; 2 Département de psychologie Université du Québec à Montréal Montréal, QC Canada; 3 Centre de recherche du Centre Intégré de Santé et de Services Sociaux de l’Outaouais Gatineau, QC Canada; 4 Département de psychologie Université de Sherbrooke Sherbrooke, QC Canada; 5 Département de psychologie Université du Québec à Trois-Rivières Trois-Rivières, QC Canada; 6 École de psychologie Université Laval Québec, QC Canada

**Keywords:** working alliance, videoconference, cognitive behavioral therapy, intolerance of uncertainty, generalized anxiety disorder, treatment, outcome, therapy, anxiety, uncertainty, telehealth

## Abstract

**Background:**

Previous meta-analyses have shown a significant relationship between working alliance and treatment outcome in general. Some studies have examined the relationship between working alliance and treatment outcome during telepsychotherapy, but to the best of our knowledge, no study has examined the mediating role of individual components of the working alliance.

**Objective:**

As part of a clinical trial of cognitive behavioral therapy (CBT) for generalized anxiety disorder (GAD) delivered by videoconference (VC), the aim of this study is to examine the mediating role of intolerance of uncertainty on the relationship between the components of the working alliance and treatment outcome.

**Methods:**

A sample of 46 adults with primary GAD received 15 sessions of CBT for GAD delivered over VC. Participants completed the measure of working alliance immediately after the fifth therapy session. The degree of change in intolerance of uncertainty (a key psychological process) was assessed from pre- to posttreatment. Treatment outcome was assessed via changes in GAD symptoms from pretreatment to the 6-month follow-up.

**Results:**

The results revealed that the therapeutic bond did not predict treatment outcome (*r*=−0.23; *P*=.12). However, agreement on therapeutic goals and tasks did predict treatment outcome (*r*=−0.42; *P*=.004 and *r*=−0.37; *P*=.01, respectively). In addition, the relationship between consensus on therapeutic tasks and treatment outcome was completely mediated by changes in intolerance of uncertainty (unstandardized β=−0.03; *r*^2^=0.12), whereas consensus relative to treatment goals had a direct impact on treatment outcome.

**Conclusions:**

These results provide a better understanding of the differential role of the components of the working alliance in telepsychotherapy as a facilitative factor for changes in key cognitive processes, leading to therapeutic change.

**Trial Registration:**

International Standard Randomized Controlled Trial Number (ISRCTN): 12662027; http://www.isrctn.com/ISRCTN12662027.

## Introduction

### Background

Anxiety disorders are among the most prevalent psychological disorders [1]. Several psychological treatments have been developed and validated for anxiety disorders [2], but the accessibility of these treatments remains limited [3]. The lack of access to available adapted services leads to several financial (eg, travel and work absenteeism) and personal (eg, time away from the family) consequences [4]. Telepsychotherapy offers an interesting solution to the above-mentioned problems in that it can limit travel costs, resulting in increased access to services from professionals specialized in empirically supported treatments. Several technologies can be used to provide remote treatment (ie, telephone, internet, and video). Videoconference (VC) can facilitate access to care because it offers an interactive communication system that provides access to verbal and nonverbal content between the patient and therapist [5-8].

Several researchers and clinicians have argued that the working alliance is a key element involved in therapeutic change [9]. The working alliance, as defined by Bordin [10], involves 3 components: (1) agreement on global treatment goals, (2) agreement on specific therapeutic tasks, and (3) the establishment of a therapeutic bond between the patient and therapist. First, the patient and therapist should share the same objectives that are addressed during treatment. Second, the consensus on tasks is improved when the patient considers that the tasks included in the therapy are logical, accessible, and relevant to the therapeutic objectives. In cognitive behavioral therapy (CBT), agreement on tasks facilitates interventions that target unhelpful thoughts and behaviors, and agreement on goals is essential to draw a shared treatment plan. Third, the bond refers to the trust and constructive attachment between the patient and therapist. Together, these 3 aspects contribute to the development of a strong and effective working alliance, as conceptualized and assessed by the Working Alliance Inventory (WAI) [11].

According to many authors, the working alliance has a considerable impact on treatment efficacy [12]. In 2 meta-analyses [9,13], the authors found that the working alliance, most commonly assessed with the WAI, predicted general treatment efficacy. It explained 5% [9] to 7.5% [13] of the variance in treatment outcome. More recently, Buchholz and Abramowitz [14] critically reviewed the literature on the working alliance during CBT for anxiety disorders. Their findings highlighted the need for more studies on the contribution of the working alliance to treatment outcome. They found 7 studies on anxiety disorders, suggesting that the global measures of alliance predicted treatment outcome, and 4 studies that did not find this result [14]. The authors found only 3 studies that investigated the specific role of the components of the working alliance. They confirmed the observation of Horvath [15] that there is a “practical vacuum in the literature.” All 3 studies (ie, 1 on posttraumatic stress disorder and 2 on obsessive-compulsive disorder) revealed that the agreement on tasks was related to treatment outcome. In summary, the data suggest that the agreement on therapeutic tasks is a component of the working alliance, which is most consistently related to therapeutic change.

In addition to the role of the working alliance, CBT models suggest that changes in cognition and behaviors as well as the ability to tolerate distress are the most important factors leading to therapeutic change [16-18]. Different therapeutic strategies, such as behavioral experiments and exposure, are suggested to inhibit maladaptive interpretations and beliefs, to change maladaptive behavioral responses, and to build a stronger ability to tolerate distress [18-20]. According to CBT models, the primary factor that explains therapeutic change is not the working alliance but rather the changes in cognition and behaviors. Working alliance is considered as a facilitative factor for changes in key processes that generate therapeutic change [21].

Some clinicians have expressed concerns that VC-based psychotherapies might hinder the establishment of a sound working alliance (refer to the study by Connolly et al [7] for a detailed review) [22]. Although several studies have examined the role of the working alliance in face-to-face therapy [13], only a handful of studies have examined the role of the working alliance in e-therapy (ie, VC, virtual reality, chat, or email) [23,24]. Overall, the results suggest that the use of VC-based psychotherapies does not interfere with the quality of the working alliance [25-27]. Although these results are encouraging, further studies are needed to understand the exact role of the working alliance and its components in telepsychotherapy. An important next step is to investigate how the 3 components of the working alliance affect treatment outcome in VC when taking into account known key treatment mechanisms. We chose to explore this question in the context of generalized anxiety disorder (GAD), which is a disorder characterized by chronic and excessive worry and anxiety that is difficult to control. GAD is a common anxiety disorder [1] and is associated with several consequences, such as higher levels of unemployment, health service use, and risk of cardiovascular disorders [28,29]. According to Roberge et al [30], approximately 80% of the people with GAD do not receive an appropriate treatment due to geographical constraints (ie, diminished availability in rural areas). Only a few studies have examined the association between the working alliance and therapeutic change for patients with GAD [14,31]. From these studies, 2 studies suggest that a strong working alliance is associated with a greater change in GAD symptoms following face-to-face CBT [31,32]. However, 2 studies did not find such a result following face-to-face CBT [33] or internet-delivered CBT [34]. The contribution of the components of the working alliance to change in GAD symptoms, especially in CBT delivered by VC, has not yet been studied. At this point, it is unclear whether any of these components can predict treatment outcome or facilitate changes in key cognitive processes.

Different cognitive behavioral models have been proposed for GAD. Each model suggests a specific vulnerability factor that contributes to the etiology and maintenance of pathological symptoms, such as cognitive avoidance [35] and metacognitive beliefs [36]. Our group has developed and validated a cognitive behavioral model of GAD that focuses on the role of intolerance of uncertainty [37]. Robichaud et al [38] defined intolerance of uncertainty as a dispositional characteristic arising from a set of catastrophic beliefs about uncertainty and its consequences. According to Robichaud et al [38], this set of beliefs leads to negative and unhelpful cognitive, behavioral, and emotional reactions in uncertainty-inducing situations (ie, situations that are novel, unpredictable, or ambiguous). Data suggest that intolerance of uncertainty is a causal risk factor for high levels of worry and GAD and that it plays a key role in the etiology of GAD [39-43]. A total of 4 randomized clinical trials support the efficacy of CBT for GAD, focusing on intolerance of uncertainty, compared with a waiting list [44,45], supportive therapy [46], and applied muscular relaxation [47]. An independent clinical trial [48] found that change in intolerance of uncertainty, as measured with the Intolerance of Uncertainty Scale (IUS) [49], mediated change in worry, whereas change in worry did not mediate change in intolerance of uncertainty. According to this model, decreases in intolerance of uncertainty play an active role in the reduction of GAD symptoms. Therefore, it is crucial to understand the variables that contribute to a greater change in key factors (eg, intolerance of uncertainty) that subsequently lead to change in symptoms (eg, worry and anxiety).

### Objectives

Using data from a clinical trial of CBT delivered by VC for GAD, the goal of this study is to gain a better understanding of the relationship between the different components of the working alliance and treatment outcome. First, we examined whether any of the 3 components of the working alliance, as perceived by the participant and as defined by Bordin [10], would predict treatment outcome. We hypothesized that, of all the components, agreement on the task would predict the treatment outcome (*Hypothesis 1*). Second, we explored whether a change in intolerance of uncertainty would mediate the relationship between the components of the working alliance and treatment outcome (*Hypothesis 2*).

## Methods

### Study Participants

Our sample consisted of 46 adults (40 women) with primary GAD participating in a randomized controlled trial (described elsewhere; refer to the studies by Watts et al [27] and Bouchard et al [50]) and allocated to receive psychotherapy delivered by VC. The mean age was 42.39 (SD 15.80) years, ranging from 20 to 74 years. The participants’ level of education varied between high school (6/46, 13%), college (14/46, 30%), and university (26/46, 56%). Participants were recruited from 5 urban areas in the province of Québec. The severity of GAD was assessed using the 9-point (0-8) Clinician’s Severity Rating (CSR) of the Anxiety Disorders Interview Schedule for the Diagnostic and Statistical Manual of Mental Disorders-IV (ADIS-IV). CSR ratings of 4 and higher correspond to the range associated with sufficient clinical severity to warrant the presence of a diagnosis. The mean severity of GAD before treatment was 5.37 (SD 1.07; range 4-7).

### Measures

The ADIS-IV [51] is a structured interview used to determine the presence and severity of several psychological disorders, such as anxiety, mood, substance use, and psychotic disorders. The ADIS-IV was used in this study to establish if participants met the diagnostic criteria of GAD for eligibility (ie, severity above 4 on the CSR). Good interrater reliability has been reported for the severity of GAD (*r*=0.72) [52].

The Penn State Worry Questionnaire (PSWQ) [53] was used to assess the GAD symptom of worry and was our measure of treatment outcome. It has 16 items that measure the tendency to worry uncontrollably and excessively. Each item was evaluated on a 5-point Likert scale. Examples of items include *My worries overwhelm me* and *Once I start worrying, I cannot stop*. The French translation of the PSWQ has excellent internal consistency (α=.82) and test-retest reliability (*r*=0.86) [54]. To measure treatment outcome, PSWQ was administered at pretreatment and at the 6-month follow-up.

The IUS [49] was our measure of the process of change (ie, our mediator). It has 27 items measuring catastrophic beliefs about uncertainty and the consequences of being uncertain. The items were evaluated on a 5-point Likert scale. Examples of items include *Uncertainty makes life intolerable* and *The smallest doubt can stop me from acting.* The IUS measures the implications of the state of uncertainty and attempts to control future events. The IUS, which was originally developed in French, has good metric proprieties. It has excellent internal consistency (α=.91) [49] and good test-retest reliability (*r*=0.78) [55]. To measure the treatment process, the IUS was administered at pretreatment and posttreatment.

The WAI [11] assesses the quality of the working alliance. The WAI has 36 items rated by the participant on a 7-point Likert scale. The higher the total score, the more the patient (respondent) perceives a good working alliance with his or her therapist. This instrument is based on the conceptualization of the working alliance and its 3 components by Bordin [10], which are measured by 3 subscales: (1) WAI-Goal (eg, *We have established a good understanding of the kind of changes that would be good for me*), (2) WAI-Task (eg, *My therapist and I agree about the things I will need to do in therapy to help improve my situation*), and (3) WAI-Bond (eg, *I believe my therapist is genuinely concerned for my welfare*). The WAI has excellent internal consistency (α=.96) [11] and acceptable test-retest reliability (*r*=0.73) [9]. In addition, the 3 subscales showed appropriate levels of intercorrelation (*r*=0.69-0.92). Like the original English version of the WAI, the French translation [56] has sound psychometric properties. Although several instruments have been developed to measure the quality of the working alliance, the WAI is the most frequently used questionnaire in both research and clinical settings [9,13]. To make an informed assessment of the quality of the working alliance without being unduly influenced by their progress in therapy [27,57], the participants completed WAI after the fifth therapy session.

### Procedure

The study was approved by the relevant ethics review boards, registered as a clinical trial, and conducted in accordance with ethical codes of conduct (eg, free and informed consent; refer to the study by Watts et al [27] for details). Participants were assessed by a team psychologist using the ADIS-IV (refer to the study by Watts et al [27] for the CONSORT [Consolidated Standards of Reporting Trials] flow chart). Those who met the eligibility criteria (n=148) were randomly assigned to 1 of the 2 conditions (ie, face-to-face CBT, n=79 or CBT delivered by VC, n=69). For this study, we only included the 46 participants assigned to the VC condition who completed treatment and had no missing values on the measures at all assessment points. Each participant was randomly assigned to 1 of the therapists for the duration of the treatment, and they never met face-to-face. They completed an individual CBT program of 15 weekly sessions based on the Intolerance of Uncertainty model, as described in the study by Robichaud et al [38]. The participants traveled to the closest city from where they live (ie, the 5 treatment centers) to receive the treatment delivered by VC through the specialized equipment that provides encrypted communication and ensures confidentiality. During each session, participants sat alone in an office, facing a television and a Tandberg Edge 95 MXP system located 2 meters away, whereas their therapist was located at a different site using a similar VC system. All units installed in clinics corresponded to the standards established for telehealth [58]. For more details on the methodology, refer to the study by Watts et al [27].

### Analytical Strategy

Pearson correlation analyses were performed between the score of each of the 3 subscales of the WAI and changes in the PSWQ (from pretreatment to the 6-month follow-up). After testing the first hypothesis, we conducted mediation analyses with bootstrapped samples (5000 samples and bias-corrected 95% CIs) using the PROCESS macro for mediation in IBM SPSS [59]. In each analysis, the predictor was the component of the WAI (the subscales that predicted change in the PSWQ), the mediator was the residualized change scores (RCSs) on the IUS (from pre- to posttreatment), and the outcome variable was the RCS on the PSWQ (from pretreatment to the 6-month follow-up). In mediation analyses, it is important to have a study design that allows for conclusions about causality. As we were interested in the role of a phenomenon occurring during psychotherapy (the working alliance) on changes that occurred over treatment, selecting appropriate time points was important for measuring change. Some overlap was unavoidable with the use of pretreatment scores to measure intolerance of uncertainty and GAD symptoms. To fully capture the change in intolerance of uncertainty, the change from pretreatment to posttreatment was used. To minimize the overlap and increase the potential of addressing causality, changes in symptoms from pretreatment to follow-up were used to measure long-term outcome. This limitation should be considered when interpreting mediation analyses.

No extreme multivariate data points were observed using Mahalanobis distance (*P*<.001). No transformation was performed on the data. The means, SDs, and ranges of the questionnaires are presented in Table 1.

**Table 1:** Descriptive statistics for all study measures during cognitive behavioral treatment for generalized anxiety disorder delivered by videoconference (N=46).

Variable	Mean (SD)	Score range
WAI-Goal^a^ at session 5	76.35 (7.94)	50-84
WAI-Task^b^ at session 5	78.30 (6.85)	54-84
WAI-Bond^c^ at session 5	75.61 (8.28)	49-84
IUS^d^ at pre-tx^e^	83.67 (20.00)	41-122
IUS at post-tx^f^	53.34 (17.78)	28-91
PSWQ^g^ at pre-tx	68.15 (6.26)	50-80
PSWQ at the 6-month follow-up	44.84 (10.46)	20-65

^a^WAI-Goal: Working Alliance Inventory, goal subscale.

^b^WAI-Task: Working Alliance Inventory, task subscale.

^c^WAI-Bond: Working Alliance Inventory, bond subscale.

^d^IUS: Intolerance of Uncertainty Scale.

^e^pre-tx: pretreatment.

^f^post-tx: posttreatment.

^g^PSWQ: Penn State Worry Questionnaire.

## Results

### Working Alliance and Treatment Outcome

The first hypothesis was that patient-perceived agreement on task would predict changes in the level of worry from pretreatment to the 6-month follow-up. The hypothesis was partially supported, as 2 of the 3 subscales of the WAI were correlated with changes in PSWQ scores. Specifically, the WAI-Goal and WAI-Task subscales significantly predicted changes in the PSWQ. However, the WAI-Bond subscale did not significantly predict changes in the PSWQ scores. Thus, both agreement on goals and agreement on tasks were associated with greater decreases in the GAD symptom of worry following a CBT delivered by VC. A correlation matrix including all variables is presented in Table 2.

**Table 2:** Correlation matrix (Pearson *r* and two-tailed *P* value) of all study measures during cognitive behavioral treatment for generalized anxiety disorder delivered by videoconference (N=46).

Variable		WAI-Goal^a^ at session 5	WAI-Task^b^ at session 5	WAI-Bond^c^ at session 5	RCS^d^ Intolerance of Uncertainty Scale (pretreatment to posttreatment)	RCS Penn State Worry Questionnaire (pretreatment to the 6-month follow-up)
**WAI-Goal at session 5**						
	*r*	1	0.87	0.73	−0.31	−0.42
	*P* value	—^e^	<.001	<.001	.03	.004
**WAI-Task at session 5**						
	*r*	0.87	1	0.63	−0.43	−0.37
	*P* value	<.001	—	<.001	.003	.01
**WAI-Bond at session 5**						
	*r*	0.73	0.63	1	−0.16	−0.23
	*P* value	<.001	<.001	—	.28	.12
**RCS Intolerance of Uncertainty Scale (pretreatment to posttreatment)**						
	*r*	−0.31	−0.43	−0.16	1	0.55
	*P* value	.03	.003	.28	—	<.001
**RCS Penn State Worry Questionnaire (pretreatment to the 6-month follow-up)**						
	*r*	−0.42	−0.37	−0.23	0.55	1
	*P* value	.004	.01	.12	<.001	—

^a^WAI-Goal: Working Alliance Inventory, goal subscale.

^b^WAI-Task: Working Alliance Inventory, task subscale.

^c^WAI-Bond: Working Alliance Inventory, bond subscale.

^d^RCS: residual change score.

^e^Not applicable.

### Mediation Analyses

The second hypothesis was that a change in intolerance of uncertainty would mediate the relationship between the working alliance and treatment outcome. Considering that we performed 2 mediation analyses, we applied Bonferroni corrections and adjusted significance levels (*P*<.025).

#### WAI-Goal Subscale

The first mediation model with the goal subscale of the WAI was not supported. The relationship between the WAI-Goal subscale and change on the IUS was not significant (unstandardized β=−0.04; *P*=.03). Changes in intolerance of uncertainty did not mediate the relationship between agreement on goals and treatment outcome (due to the Bonferroni correction). However, it is important to note that the relationship was close to being significantly supported.

#### WAI-Task Subscale

**Figure 1:**
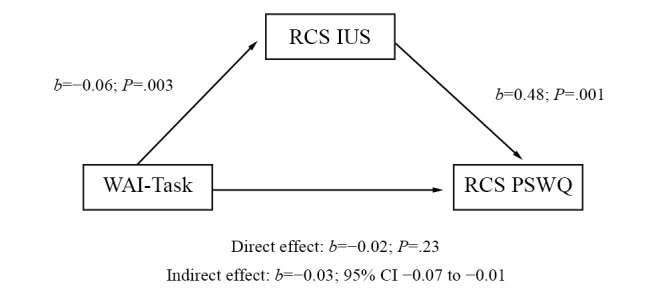
Summary of the mediation analysis with the Working Alliance Inventory task subscale during cognitive behavioral treatment for generalized anxiety disorder delivered by videoconference (N=46). *b*: unstandardized beta coefficient; IUS: Intolerance of Uncertainty Scale; PSWQ: Penn State Worry Questionnaire; RCS: residual change score; WAI-Task: Working Alliance Inventory, task subscale.

The second mediation model, with the task subscale of the WAI, was supported. Scores on the WAI-Task subscale significantly predicted change on the IUS. Moreover, change on the IUS significantly predicted change on the PSWQ. Once the indirect effect was taken into account, the direct effect of the WAI-Task subscale on change on the PSWQ was no longer significant. The WAI-Task subscale had a significant indirect effect on change on the PSWQ (pretreatment to the 6-month follow-up), which was mediated by the change on the IUS (pretreatment to posttreatment). The indirect effect had a medium effect size (*r*^2^=0.12). Changes in intolerance of uncertainty completely mediated the relationship between agreement on task and the change in the GAD symptom of worry. The results are shown in Figure 1.

## Discussion

### Principal Findings

The aim of this study is to provide a better understanding of the mechanisms of change during CBT for GAD delivered by VC. We examined the mediating role of uncertainty intolerance in the relationship between the selected components of the working alliance and treatment outcome. The results showed that (1) the therapeutic bond does not significantly predict treatment outcome; (2) agreement on therapeutic goals predicts treatment outcome but does not predict change in intolerance of uncertainty; and (3) agreement on therapeutic tasks predicts treatment outcome, and this effect is completely mediated by change in intolerance of uncertainty.

First, the results showed that a stronger therapeutic bond is not related to a greater change in symptoms. This result is in line with previous studies that investigated the 3 components of the working alliance during face-to-face CBT for several psychological disorders, including anxiety disorders [14]. Watts et al [27] compared the working alliance in VC and in face-to-face in our sample and found no evidence that it was poorer in VC (it was actually significantly stronger), suggesting that our findings are not simply the result of an impoverished therapeutic bond in VC. Bouchard et al [60] also found no predictive impact of the therapeutic bond on treatment outcome, in VC or in face-to-face, for patients with panic disorder and agoraphobia. The results of this study add to a growing body of evidence suggesting that the working alliance predicts treatment outcome because of the agreement on goals and tasks. In their review of the VC literature, Simpson and Reid [61] reported that several studies support the notion that VC treatments can generate a strong therapeutic bond right from the onset of treatment [26,62,63]. For example, Germain et al [26] found no significant difference in the quality of the bond between a VC-based treatment and a face-to-face treatment for posttraumatic stress disorder. Our results add to the literature by providing data that are specific to GAD and by showing that the bond does not predict change in outcome. Thus, although a strong therapeutic bond between the patient and therapist is considered to be a prerequisite for successful CBT [21], it does not appear to be a predictor of improvement.

A second interesting result from this study is that agreement on therapeutic goals predicted treatment outcome. This was somewhat unexpected because studies conducted with other disorders have produced conflicting findings [64,65]. Of note, the relationship between agreement on goals and change in intolerance of uncertainty was nonsignificant (due to the Bonferroni correction). A posteriori power analysis showed that our study was not optimally statistically powered; at least 126 participants would be required to test the mediation model with a power of 0.80 and *without* the use of a Bonferroni correction for type 1 error [66]. The absence of a significant relationship is slightly surprising because, in CBT, agreement on general goals is expected to be related to core therapeutic processes. Although agreement on goals was related to treatment outcome but not to the core mechanism of change in CBT of GAD, the pattern of results led to a mediation effect of intolerance of uncertainty that was close to reaching the threshold of statistical significance. Overall, the findings may be explained by the specific nature of GAD and its treatment. Many patients with GAD seek treatment to gain more control over their anxiety and gain more certainty. However, to be truly effective, the treatment must not target anxiety but *the tolerance* of uncertainty [46,47]. This slight nuance in goals may lead to a weaker fit with uncertainty. These results suggest that the need to be in agreement with the goals of the treatment of GAD is important to treatment success and independent of strengthening tolerance of uncertainty. As agreement on goals correlated strongly with agreement on tasks, addressing the (lack of) usefulness of worrying and the importance of tolerating uncertainty must not be neglected. Future studies should investigate the mediational relationship between the agreement on goals and changes in intolerance of uncertainty with a larger sample.

Third, our results reveal that intolerance of uncertainty mediates the relationship between agreement on tasks and changes in symptoms when CBT is delivered by VC. This result is relevant to CBT in general, as it shows that agreement on therapeutic tasks leads to greater change in beliefs about uncertainty, which then leads to a decrease in GAD symptoms. Our results are also relevant to CBT delivered by VC for GAD by showing that a component of the working alliance acts as a facilitative factor for change in the key process of the disorder (in this case, intolerance of uncertainty), which ultimately leads to therapeutic change. Several studies have suggested that building a working alliance is not an end in and of itself in the treatment of anxiety disorders but rather a basis upon which patients and their therapists can work to reach changes in core beliefs that maintain the disorder [26,61,62]. However, this study is the first to support this hypothesis with data from psychotherapy delivered by VC. Moreover, it is the first study to address the working alliance in the field of VC, which tests a mediation effect for a cognitive change variable. Our results suggest that when patients perceive the tasks in therapy as logical, accessible, and relevant to the therapeutic objectives, they may be more prone to tolerate distress to attain clinical change in maladaptive beliefs and behaviors. Overall, VC does not seem to be a barrier to the establishment of a sound working alliance or successful therapy.

### Strengths and Limitations

It is important to highlight some of the limitations of this study. First, the data were obtained using self-report questionnaires completed by the participants. We did not use a measure of the therapist’s impression of the working alliance, and we did not obtain an independent clinician’s impression of GAD symptoms. Second, the measure of the working alliance was completed on only 1 occasion, as opposed to several times over the course of therapy (in which case, an aggregated score could have been used). The content of the specific session that immediately preceded the completion of the WAI may have had an impact on the perception of the working alliance, whereas a mean score aggregated over several sessions might be less unstable. Finally, the change scores for intolerance of uncertainty (pretreatment to posttreatment) and for GAD worry (pretreatment to 6-month follow-up) overlap in terms of their timing; both change scores use the pretreatment time point. To overcome this limitation, we could have examined changes in GAD symptoms from posttreatment to the 6-month follow-up and changes in intolerance of uncertainty from pre- to posttreatment. However, such an attempt to avoid using the same time point would have resulted in measuring fluctuations in worry *after* therapy and not during therapy. Given that our research questions focus on the change in symptoms occurring during therapy and not on the maintenance of therapeutic gains after treatment, this limitation was unavoidable.

Simpson and Reid’s [61] review of the role of the working alliance during VC-based treatment suggests several benefits regarding the use of this technology. First, despite the physical distance, patients and therapists report feeling that they are in the same room and that they are absorbed by the interaction. Different authors have argued that the feeling of telepresence can facilitate the establishment of a sound therapeutic bond and of collaborative goals specific to the working alliance [24,67]. Furthermore, VC can reduce the feeling of being intimidated or pressured while increasing the feeling of control over the treatment. Simpson and Reid [61] also concluded that across the various studies identified, the working alliance is generally strong, although some studies have found it to be lower [23]. Nevertheless, these authors suggest several factors that may influence the quality of the working alliance and that deserve to be studied more thoroughly, such as the level of telepresence, therapist competence, or patient attitudes and beliefs.

### Conclusions

To summarize, the results of this study document the role of the different components of the working alliance in CBT delivered by VC. This study also documents the mechanisms of change during treatment for GAD. Our results suggest that the 2 components of the working alliance predict treatment outcome. Agreement on goals has a direct impact on changes in symptoms, whereas agreement on tasks has an indirect effect via changes in intolerance of uncertainty. Our results suggest that it is important to ensure that the patient and therapist agree on the goals and tasks to be performed in therapy to ensure optimal treatment success. Our results also highlight the role of the working alliance in understanding the mechanisms of change in GAD. Future studies should examine whether the relationship between working alliance and treatment outcome is, in fact, due to agreement on the goals and tasks to be performed in therapy, rather than on the therapeutic bond.

## Abbreviations

ADIS-IV: anxiety disorders interview schedule for the Diagnostic and Statistical Manual of Mental Disorders-IV
CBT: cognitive behavioral therapy
CSR: Clinician’s Severity Rating
GAD: generalized anxiety disorder
IUS: Intolerance of Uncertainty Scale
PSWQ: Penn State Worry Questionnaire
RCS: residualized change score
VC: videoconference
WAI: Working Alliance Inventory
